# The microbiome in *PTEN* hamartoma tumor syndrome

**DOI:** 10.1530/ERC-17-0442

**Published:** 2017-12-12

**Authors:** Victoria Byrd, Ted Getz, Roshan Padmanabhan, Hans Arora, Charis Eng

**Affiliations:** 1 Genomic Medicine InstituteLerner Research Institute, Cleveland Clinic, Cleveland, Ohio, USA; 2 Case Western Reserve University School of MedicineCleveland, Ohio, USA; 3 Glickman Urological & Kidney InstituteCleveland Clinic, Cleveland, Ohio, USA; 4 Taussig Cancer InstituteCleveland Clinic, Cleveland, Ohio, USA; 5 Department of Genetics and Genome SciencesCase Western Reserve University School of Medicine, Cleveland, Ohio, USA; 6 CASE Comprehensive Cancer CenterCase Western Reserve University School of Medicine, Cleveland, Ohio, USA

**Keywords:** microbiome, breast cancer, endometrial cancer, thyroid cancer, inherited cancer syndromes

## Abstract

Germline *PTEN* mutations defining PTEN hamartoma tumor syndrome (PHTS) confer heritable predisposition to breast, endometrial, thyroid and other cancers with known age-related risks, but it remains impossible to predict if any individual will develop cancer. In the general population, gut microbial dysbiosis has been linked to cancer, yet is unclear whether these are associated in PHTS patients. In this pilot study, we aimed to characterize microbial composition of stool, urine, and oral wash from 32 *PTEN* mutation-positive individuals using 16S rRNA gene sequencing. PCoA revealed clustering of the fecal microbiome by cancer history (*P* = 0.03, *R* ^2^ = 0.04). Fecal samples from PHTS cancer patients had relatively more abundant operational taxonomic units (OTUs) from family Rikenellaceae and unclassified members of Clostridia compared to those from non-cancer patients, whereas families Peptostreptococcaceae, Enterobacteriaceae, and Bifidobacteriaceae represented relatively more abundant OTUs among fecal samples from PHTS non-cancer patients. Functional metagenomic prediction revealed enrichment of the folate biosynthesis, genetic information processing and cell growth and death pathways among fecal samples from PHTS cancer patients compared to non-cancer patients. We found no major shifts in overall diversity and no clustering by cancer history among oral wash or urine samples. Our observations suggest the utility of an expanded study to interrogate gut dysbiosis as a potential cancer risk modifier in PHTS patients.

## Introduction

Individuals with germline *PTEN* mutations have an inherited cancer syndrome known as *PTEN* hamartoma tumor syndrome (PHTS), which is characterized by increased risk of endocrine cancers including 85% lifetime cancer risk for the female breast, 35% thyroid and 28% endometrium; non-endocrine cancers are also components of the syndrome and include renal cancer (33% lifetime risk), colorectal (9%) and melanoma (6%) (Eng 2001, Tan *et al*. 2012). These data provide evidence for *PTEN*-enabled cancer risk assessment, surveillance and medical management for PHTS patients as a group. However, there is currently no way to predict if any one individual harboring a *PTEN* mutation will develop cancer, and if so, which cancer(s). There is, therefore, a need to ascertain clinically significant risk factors for cancer at an individual level in PHTS.

Cancer susceptibility is influenced by interactions between environmental and genetic factors; however, our understanding of which environmental factors influence cancer risk is incomplete. While the environmental component of a few cancers can be largely attributed to a single carcinogen—for example, lung cancer and tobacco smoking—this represents the exception, as most environmental contributors to cancer risk remain to be characterized (Bultman 2014). The human microbiome has recently received much attention as a potential modifier of cancer risk in the general population (Plottel & Blaser 2011). Breast cancer has been linked to dysbiosis, or perturbation of the normal microbial composition, of the breast and oral cavity (Xuan *et al*. 2014, Freudenheim *et al*. 2016). This association may be at least partly explained by altered microbial metabolism of estrogen in the dysbiotic microbiome (Flores *et al*. 2012, Fuhrman *et al*. 2014). Dysbiosis of the gut has been implicated in colorectal cancer (CRC), and alterations in the urinary microbiota have been linked to urothelial carcinoma (Louis *et al*. 2014, Xu *et al*. 2014). Proposed mechanisms for these links include injection of direct effectors into host cells, induction of a pro-inflammatory microenvironment and altered host–microbiota interactions leading to activation of key cancer-promoting pathways like STAT3 and NF-κB (Rajagopala *et al*. 2017).

No study has yet investigated whether microbial dysbiosis of the gut, urinary tract or oral cavity is associated with heritable cancer specifically in patients with germline *PTEN* mutations. An important question is whether differences in the oral, urinary tract and/or gut microbiota of individuals with germline *PTEN* mutations are associated with the neoplastic outcome.

We therefore sought to conduct a hypothesis-generating study with the broad view that the microbiome might be a modifier of *PTEN-*related disease risk. Specifically, we wished to explore whether the microbial communities of the gut, oral cavity and urinary tract would differ in diversity and overall composition between PHTS patients with and without component cancers.

## Materials and methods

### Patient enrollment and sample collection

With approval from our Institutional Review Board for Human Subjects’ Protection and after written informed consent, we contacted the subset of patients in our longitudinal Molecular Mechanisms of Cancer study (protocol 8458-PTEN) with identified pathogenic germline mutations in the *PTEN* gene. From this cohort, we enrolled 17 individuals with a history of component cancers and 15 without a history of cancer. History of cancer was determined by self-reporting and confirmed via medical record review. Demographics, clinical history and diet and lifestyle information were collected prospectively through completion of a questionnaire at the time of enrollment (Supplementary Fig. 1, see section on supplementary data given at the end of this article).

From each patient, we obtained a midstream clean-catch urine specimen, a saline oral rinse sample, and a stool sample at the time of written consent from patients seen in our multidisciplinary *PTEN* clinic and by mail from patients not seen in clinic.

Urine was centrifuged at 600 ***g*** for 10 min. Oral rinse supernatant was centrifuged at 3000 ***g*** for an additional 15 min. After decanting the supernatant, the pellet was frozen and stored at −80°C until nucleic acid extraction. Stool samples were aliquoted into microcentrifuge tubes and stored at −80°C until nucleic acid extraction.

### DNA extraction

Total DNA was extracted from urine and oral rinse pellets, and from fecal specimens, using PowerViral RNA/DNA Isolation kit according to the manufacturers’s protocol (Mo Bio Laboratories, Carlsbad, CA, USA) with minor modifications. Pellets were resuspended in 650 µL MoBio PV1 solution with ß-mercaptoethanol, and then transferred to PowerViral glass bead tubes and warmed at 55°C for 10 min. Samples were homogenized using the TissueLyser LT (Qiagen) at 25 Hz for 10 min and centrifuged at 13,000 ***g*** for 1 min, after which supernatants were transferred to a clean 2 mL collection tube with 150 µL of solution PV2 and incubated at 4°C for 5 min. Lysates were centrifuged at 13,000 ***g*** for 1 min, and supernatants transferred to a clean 2.2 mL tube with 600 µL of solutions PV3 and PV4 and vortexed, after which 625 µL of supernatant was repeatedly loaded onto a spin filter and centrifuged at 13,000 ***g*** for 1 min until all supernatant was loaded onto the filter. 600 µL each of solutions PV5 and PV6 were added, with 1 min of centrifugation after each, discarding flow-through; tubes were then centrifuged for 2 min before spin filter basket was placed into a clean tube and DNA eluted in 100 µL of RNAse-free water.

### 16S rRNA gene sequencing

Bacterial 16S rRNA gene amplification and library construction was performed according to the 16S Metagenomic Sequencing Library Preparation guide from Illumina (Forest City, CA, USA) with minor modifications. All beads, tubes and non-enzymatic reagents were treated with UV light for 30 min prior to use (Tamariz *et al*. 2006). Briefly, total DNA was PCR-amplified using primers targeting the 16S V3 and V4 region (Illumina) (Klindworth *et al*. 2013) under the following conditions: 95°C for 5 min, followed by 35 cycles of 95°C for 30 s, 56°C for 30 s, 72°C for 30 s and a final extension of 72°C for 10 min. The resulting 16S rDNA amplicons were run on a 1% agarose gel, size-selected at 450–500 bp and gel-purified using QIAquick Gel Purification kit (Qiagen). A second round of PCR was performed to add Nextera XT indices (Illumina) to purified amplicons. Indexed PCR products were purified with Ampure XP beads (Beckman Coulter, Brea, CA, USA) and quantified with Qubit dsDNA system (ThermoFisher Scientific). Samples were then normalized and pooled into sequencing libraries at 20 nM for oral wash and fecal samples and 1 nM for urine samples, then validated on a Bioanalyzer DNA 1000 chip (Agilent) and sequenced on the Illumina MiSeq with a V3 reagent kit at the Case Western Reserve University Genomics Core Facility.

### Bioinformatic analysis

Paired-end reads, which were 250 bp in length, were merged with FLASH (Magoč & Salzberg 2011). Low-quality reads (Phred <20) were filtered out using the split_libraries.py command in QIIME (version 1.9) (Caporaso *et al*. 2010). A hybrid sequencing analysis methodology was adopted, in which preprocessing was performed in QIIME and open-reference operational taxonomic unit (OTU) picking was implemented within MICCA (Albanese *et al*. 2015). Vsearch (version 1.9.5) (Rognes *et al*. 2016) was used to cluster sequences with a threshold of 97% similarity, and representative sequences were classified using RDP classifier (version 2.11) (Wang *et al*. 2007). Multiple sequence alignment was performed using MUSCLE (version 3.8.31) (Edgar 2004) against the Greengenes database (version 13.8) (DeSantis *et al*. 2006), filtered at 97% similarity, and FastTree (version 2.1.8) was used for phylogenetic tree construction (Price *et al*. 2009). Taxa represented in fewer than 5% of total samples in the group were then discarded. Rarefaction to 1000 reads per sample for fecal, 365 for urine and 1449 for oral wash samples was performed to reduce sampling heterogeneity, and computation of alpha (Shannon diversity index) and beta diversity measures (unweighted UniFrac distances) was performed with QIIME.

### Statistics

Comparison of continuous and categorical demographics/clinical factors of cancer vs non-cancer samples was performed using two-sided Student’s *t*-test. Two-sided Student’s *t*-test was used to compare Shannon index, and distance matrices were compared using the Adonis statistical method, which is based on the nonparametric analysis of variance (ANOVA) family of statistical methods and uses *F*-tests based on sequential sums of squares from permutations on weighted and unweighted UniFrac distance matrices, with the null hypothesis that there is no difference in community structure between groups. To compare relative abundances of taxa between different categorical variables, Welch’s *t*-test or the Kruskal–Wallis test was used.

To identify taxa that were differentially abundant in cancer vs non-cancer groups, *de novo* OTUs were removed, and the remaining OTUs were input into PICRUSt (Langille *et al*. 2013) and LEfSe (Segata *et al*. 2011). This algorithm performed nonparametric statistical testing of whether individual taxa differed between the class cancer vs non-cancer, and the sub-class sex, and ranked differentially abundant taxa by their linear discriminant analysis (LDA) log-score. Differentially abundant taxa that were statistically significant using an alpha of 0.05 and exceeded an LDA log-score of ±2 were visually represented on cladograms and box plots. To determine differences in predicted functional metagenomes between cancer vs non-cancer samples, open-reference OTU tables generated by QIIME/MICCA were associated with clinical/demographic data and input into PICRUSt and LEfSe via the Huttenhower Lab Galaxy Server (Blankenberg *et al*. 2010).

All statistical tests were two-sided, with *P* < 0.05 considered statistically significant. All analyses were conducted and graphs created in JMP Pro 13 (SAS Institute, Cary, NC, USA) or R packages* VEGAN* (Dixon 2003) and* phyloseq* (McMurdie & Holmes 2013). Plots were made with QIIME and *ggplot2* (Wickham 2009).

### Data availability

The datasets generated and analyzed during the current study are available from the corresponding author on reasonable request.

## Results

### Study population

A total of 32 unrelated PHTS patients were enrolled in this study, 17 of whom had a history of cancer and 15 of whom had no history of cancer. All patients returned a questionnaire assessing demographic and clinical characteristics.

Twelve of the 17 cancer patients had a history of breast cancer (71%), seven of thyroid (41%), five of kidney (29%), three of skin (18%), two of endometrial (12%) and one of colon cancer (6%). Five had a history of another type of cancer; all five also had a history of at least one component cancer of PHTS. PHTS patients with a history of cancer had a higher mean age than non-cancer PHTS patients (56 vs 34, *P* = 0.0002). There was a higher proportion of females among those with cancer than those without (77% vs 40%, *P* = 0.04). These findings reflect the age and sex distribution of our larger cohort of patients with pathogenic germline *PTEN* mutations described by Tan *et al*. (2011). Other variables assessed in the questionnaire, including BMI, diet, alcohol consumption, smoking history and antibiotic use, did not differ significantly between PHTS patients with and without cancer (Table 1).

**Table 1:** Demographic characteristics of study PHTS patients with and without cancer history.

Variable	Cancer (*N* = 17)	Non-cancer (*N* = 15)	*P* value
Age (years)	56 ± 12	34 ± 18	0.0002
Sex			
Female	13 (77)	6 (40)	0.04
Male	4 (24)	9 (60)	
BMI	31 ± 12	31 ± 9	0.4
Race			0.2
White	17 (100)	13 (87)	
Black	0 (0)	1 (7)	
Multiracial	0 (0)	1 (7)	
Smoking history			0.3
Yes	3 (18)	1 (7)	
No	14 (82)	14 (93)	
Alcohol use			0.1
Yes	11 (65)	6 (40)	
No	6 (35)	9 (60)	
Antibiotic use (past year)			0.5
Yes	7 (41)	5 (33)	
No	10 (59)	10 (67)	
Diet			0.7
Western	12 (71)	13 (87)	
Mediterranean	1 (6)	1 (7)	
Low-carb	1 (6)	0 (0)	
Paleo	1 (6)	0 (0)	
Other	2 (12)	0 (0)	
Unknown	0 (0)	1 (70)	
Mode of delivery			0.2
Vaginal	12 (71)	7 (47)	
C-section	3 (18)	6 (40)	
Unknown	2 (12)	2 (13)	

Values are presented as means ± s.d. or number (percent).

### Fecal microbiome

One patient from the non-cancer group did not contribute a fecal sample; thus, 31 fecal samples were used for the initial analysis. Grouping reads retrieved from 16s rRNA gene amplicon sequencing at 97% similarity resulted in the identification of 5888 bacterial operational taxonomic units (OTUs). After chloroplast sequences and low-prevalence (found in less than 5% of all fecal samples) OTUs were removed, 4211 OTUs remained. Overall, 10 bacterial phyla were detected in fecal samples, with Firmicutes being the most dominant (84.6%), followed by Bacteroidetes (11.4%). Other phyla were present at lower levels, including Proteobacteria (1.8%) and Actinobacteria (1.6%), and six other phyla at marginal levels (affiliated with less than 1% of sequences).

Depth of coverage for fecal samples was set to 1000 reads per sample based on leveling off of Shannon indices. Due to this cut-off, 2 of the 31 samples were excluded from further analyses; thus, 16 samples from PHTS patients with a history of cancer and 13 from PHTS patients without a history of cancer were used for the final analysis.

**Figure 1:**
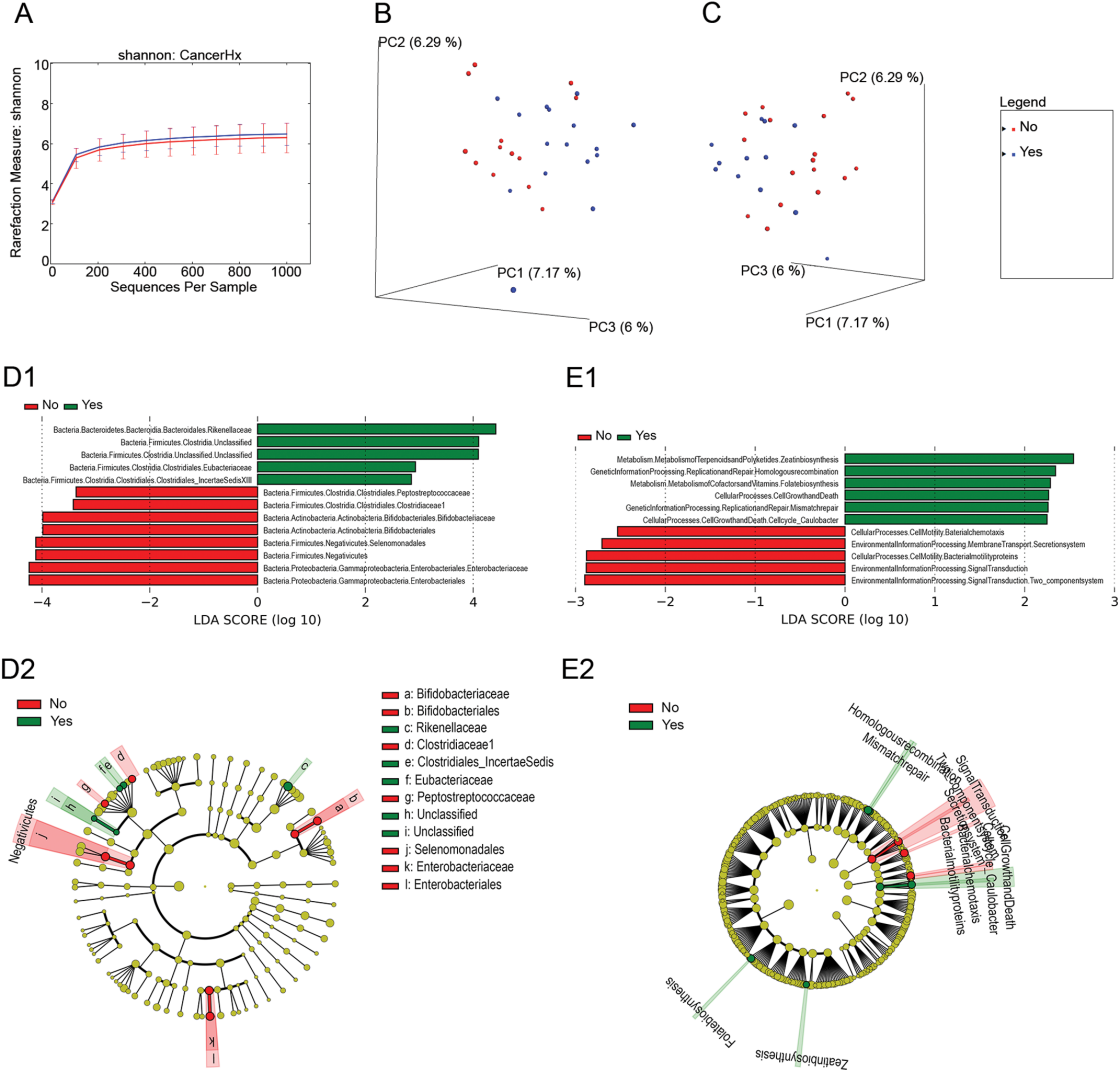
The microbiome of fecal samples is distinct between PHTS patients with and without a history of cancer. (A) Rarefaction curves comparing alpha diversity by Shannon index of fecal microbiome from patients with and without a history of cancer. (B and C) Ordination plots showing the clustering pattern of samples from patients with and without a history of cancer (B) and with and without a history of breast cancer (C) based on unweighted UniFrac distance. (D1 and D2) LEfSe differential abundance analyses of the microbiome between fecal samples from patients with (green) and without (red) cancer (D1). A cladogram demonstrates the phylogenetic relationships between differentially abundant taxa (D2). (E1 and E2) The differential KEGG pathways as revealed by PiCRUSt analysis. Clades and KEGG pathways in these graphs were considered differentially abundant if alpha > 0.05 and if the LDA log-score exceeded ±2.

Mean alpha (within-sample) diversity as measured by the Shannon index (H) was not significantly different among samples from patients with cancer (*H* = 6.5 ± 0.6) than samples from those without cancer (*H* = 6.3 ± 0.7; *P* = 0.5) (Fig. 1A). Shannon indices did not differ significantly by age (grouped by decade), sex, antibiotic use or any other clinical or demographic variable.

Principal coordinates analysis (PCoA) was used to evaluate differences in overall bacterial taxa composition in fecal samples from PHTS patients with cancer vs those without cancer. We found that samples from patients with and without a history of cancer clustered separately on unweighted UniFrac PCoA (*P* = 0.03, *R* ^2^ = 0.04; Fig. 1B). We also found that samples from patients with breast cancer clustered distinctly on unweighted UniFrac from samples from all other patients, including those with other types of cancer (*P* = 0.02, *R* ^2^ = 0.05; Fig. 1C). Finally, we observed clustering by antibiotic use on unweighted UniFrac, but this was of borderline significance (*P* = 0.05, *R* ^2^ = 0.04). However, there was no significant difference in antibiotic use between PHTS patients with and without cancer in this study. There was no significant clustering by sex, nor by any other clinical or demographic variable.

Next, we performed a LDA comparison of relative abundances with LEfSe (Fig. 1D). This revealed a relative increase in abundance of families Rikenellaceae, Eubacteriaceae, two unclassified Clostridia and Clostridiales bacterium S5-A14a (also known as Clostridiales Family XIII Incertae Sedis), among fecal samples from PHTS patients with a history of cancer relative to patients without a history of cancer. Meanwhile, we observed that families Peptostreptococcaceae, Clostridiaceae 1, Bifidobacteriaceae, Enterobacteriaceae and unclassified Bifidobacteriales, Selenomodales, Enterobacteriales and Negativicutes were relatively more abundant among fecal samples from non-cancer patients compared to cancer patients.

Finally, metagenome functional content was predicted using PICRUSt and group comparisons were performed with LEfSe, revealing relatively increased predicted expression of pathways involved in folate and zeatin biosynthesis, genetic information processing (homologous recombination and mismatch repair), and cell growth and death among fecal samples from patients with a history of cancer. Meanwhile, there was predicted enrichment of pathways for signal transduction, membrane transport and bacterial motility proteins and chemotaxis among samples from patients with no history of cancer (Fig. 1E).

### Oral wash microbiome

Oral wash samples yielded 1223 bacterial OTUs. After chloroplast sequences and low-prevalence OTUs were removed, 884 OTUs remained. Ten bacterial phyla were detected in oral wash samples. Firmicutes was most dominant (46.2%), followed by Bacteroidetes (19.6%), Proteobacteria (12.0%), Actinobacteria (10.6%), Fusobacteria (5.9%), Candidatus Saccharibacteria (2.5%), Spirochaetes (1.9%) and three marginal phyla.

Two cancer patients and one non-cancer patient did not provide oral wash samples; thus, 15 samples from PHTS patients with cancer and 14 from patients without cancer were used in the analysis. Depth of coverage for oral wash samples was set to 1449 reads per sample based on leveling off of Shannon indexes. No samples were excluded due to this cut-off.

**Figure 2:**
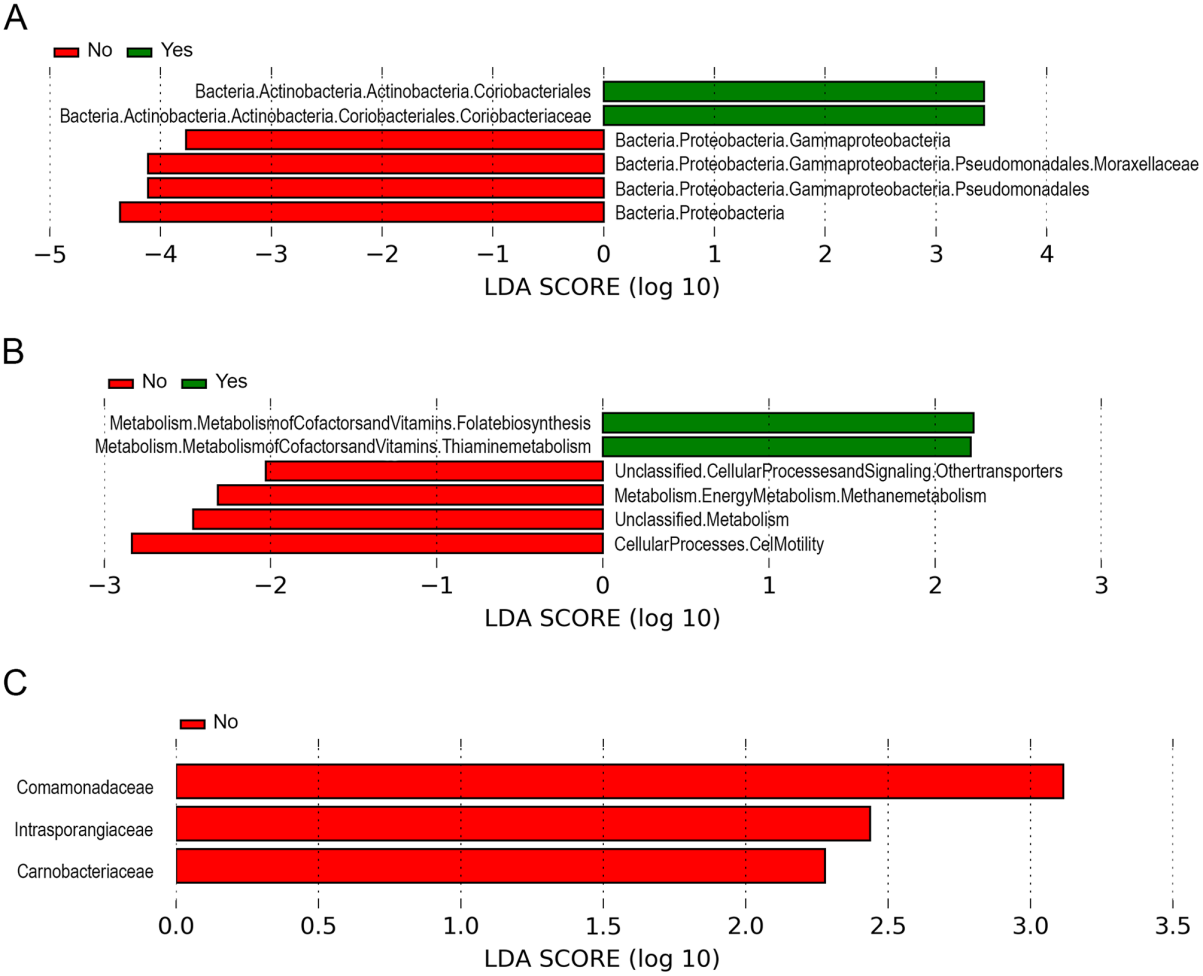
The microbiome of oral wash and urine samples varies in PHTS patients with and without a history of cancer. (A) LEfSe differential abundance analyses of the oral microbiome from patients with (green) and without (red) cancer. (B) The differential KEGG pathways of the oral microbiome from patients with (green) and without (red) cancer as revealed by PiCRUSt analysis. (C) LEfSe differential abundance analyses of the urinary microbiome between samples from patients with (green) and without (red) cancer. KEGG pathways in these graphs were classified as differentially abundant if alpha > 0.05 and if the LDA log-score exceeded ±2.

We did not find significant differences in alpha diversity in samples from PHTS patients with vs without cancer, nor in any other clinical or demographic variable. We also did not find clustering on weighted or unweighted PCoA by cancer history (Supplementary Fig. 2), sex or any other variable. LDA comparison of relative abundances with LEfSe was performed next (Fig. 2A), revealing relatively increased abundance of order Coriobacteriales, including family Coriobacteriaceae among samples from cancer patients, in contrast to the relative enrichment of family Moraxellaceae and unclassified members of Gammaproteobacteria and other Proteobacteria among samples from non-cancer patients. In addition, functional metagenome prediction (Fig. 2B) revealed increased expression of pathways involved in metabolism of vitamins and cofactors, including folate biosynthesis and thiamine metabolism, among samples from patients with cancer; pathways for cell motility, transporters and methane metabolism were predicted to be increased among samples from patients without cancer.

### Urine microbiome

Urine samples yielded 4751 bacterial OTUs. After removal of chloroplast sequences and low-prevalence OTUs, 1578 OTUs remained. Eleven bacterial phyla were detected: Firmicutes was most dominant (58.8%), then Bacteroidetes (16.4%), Actinobacteria (10.7%), Proteobacteria (10.5%), Fusobacteria (1.8%) and six marginal phyla.

One cancer patient and three non-cancer patients did not provide urine samples; thus, 16 samples derived from patients with cancer and 12 from patients without cancer were used in the final analysis. Depth of coverage for urine samples was set to 365 reads per sample based on leveling off of Shannon indices. No samples were excluded due to this cut-off.

Mean alpha diversity was not significantly different in samples from patients with vs without cancer. Alpha diversity was significantly higher in samples from male (*H* = 6.6 ± 1.1) vs female patients (*H* = 3.6 ± 1.8; *P* = 0.001). Urine samples from PHTS patients did not cluster significantly by cancer history on unweighted UniFrac PCoA (Supplementary Fig. 3A). However, we did observe significant clustering by sex (*P* = 0.001, *R* ^2^ = 0.08) (Supplementary Fig. 3B). We did not observe significant clustering by any other variable.

LDA comparison of relative abundances (Fig. 2C) revealed relatively increased abundance of families Comamonadaceae, Intrasporangiaceae and Carnobacteriaceae among patients without cancer (no families were relatively more abundant in non-cancer patients). Functional metagenome prediction revealed several differentially expressed pathways according to cancer status (Fig. 2C); however, all of these could be accounted for by differences in the predicted functional metagenome according to sex, with the exception of the predicted increased expression of fatty acid biosynthesis pathways among urine samples derived from non-cancer PHTS patients.

## Discussion

This investigation was designed as a pilot study to explore whether microbiota from different bodily sites of individuals carrying germline *PTEN* mutations would differ by cancer phenotype. We found that fecal microbiota yielded structural segregation (unweighted UniFrac distance) according to the presence or absence of cancer history. However, there was no difference in overall diversity among fecal samples from cancer patients compared to those from PHTS patients without cancer. Comparison of oral wash and urine samples of PHTS patients with vs without cancer revealed no major shifts in diversity or community structure.

Consistent with previous reports, serving as positive controls, we found that Firmicutes and Bacteroidetes were by far the most dominant phyla among human gut microbiota (Tap *et al*. 2009, Arumugam *et al*. 2011), and that Firmicutes predominated in both the oral cavity and the male and female urinary tract (Lewis *et al*. 2013, Wang *et al*. 2017).

We did not find any significant difference in alpha diversity in fecal samples from PHTS patients with compared to those without component cancers, in contrast with previous reports of decreased diversity in cancer patients (Ahn *et al*. 2013). It is well documented that gut microbiota of patients with sporadic, or non-*PTEN* related, breast and colorectal cancer compared to healthy controls are structurally distinct (Wang *et al*. 2012, Goedert *et al*. 2015, Flemer *et al*. 2017). In PHTS patients, we also found structurally distinct gut microbial communities in those with vs without cancer. While exposures related to cancer treatment may influence microbiome composition, the wide variety of combinations of chemotherapy, radiation and surgery to which PHTS cancer patients may have been exposed makes it unlikely that this was a major confounding variable. Gut microbial communities were also distinct among PHTS patients with breast cancer compared to those without breast cancer. It is possible that the gut microbiome exerts a carcinogenic effect on the breast tissue via its role in estrogen metabolism; future microbiome studies could include an analysis of urine estrogen levels in PHTS patients with and without breast cancer to investigate this possibility.

Among fecal samples from cancer patients compared to non-cancer patients, we report relatively increased abundance of family Rikenellaceae, whose members are gram-negative, non-motile anaerobes (Graf 2014) reported to be enriched in mice consuming a high-fat diet (Kim *et al*. 2012) and diabetic mice (Geurts *et al*. 2011). High levels of endogenous estrogens are a risk factor for breast cancer (Fuhrman *et al*. 2012), and obesity is a determinant of non-ovarian systemic estrogen levels and an independent risk factor for breast cancer, the most component cancer of PHTS (Cleary & Grossmann 2009). There is a known association of germline *PTEN* mutations with increased adiposity and paradoxically enhanced insulin sensitivity (Pal *et al*. 2012). In light of the many growth pathways that play dual roles in carcinogenesis and the development of obesity, it is plausible that Rikenellaceae may modulate signaling down the PI3K/AKT pathway that is constitutively activated in PHTS to modify risk for both cancer and obesity.

Estrogen metabolism in the liver results in the excretion of conjugated estrogens into the bile and eventually the gut, where they are deconjugated and reabsorbed into circulation to a variable degree. Flores and coworkers demonstrated that four Clostridia taxa in fecal samples from men and post-menopausal women were associated with non-ovarian urine estrogen levels (2012). This could provide a mechanism for increased cancer risk independent of BMI. Consistent with this observation, we observed relatively increased abundance of unclassified Clostridia in fecal samples from PHTS patients with cancer compared to those without cancer.

Peptostreptococcaceae, whose members are motile anaerobes, has been reported to be relatively overrepresented in the gut microbiome of colorectal cancer patients (Ahn *et al*. 2013). In contrast, we observed a relative underrepresentation of Peptostreptococcaceae among fecal samples from PHTS patients with cancer compared to those without cancer. Clostridiaceae 1 was also underrepresented in fecal samples from PHTS patients with cancer; members of this family include pathogens such as *Clostridium perfringens*. In addition, members of Enterobacteriales including Enterobacteriaceae were relatively more abundant among fecal samples from non-cancer compared to cancer patients. Enterobacteriaceae has been implicated in intestinal inflammation (Morgan *et al*. 2012). According to mouse studies, inflammation driven by members of the Enterobacteriaceae family is necessary but not sufficient for carcinogenesis (Arthur *et al*. 2012). Given the immune dysregulation in PHTS patients and defects in mucosal B-cell homeostasis (Heindl *et al*. 2012, Chen *et al*. 2017), it is tempting to speculate that dysbiosis-associated inflammation could contribute to PHTS-associated cancer risk. It is almost certain that some non-cancer PHTS patients in this study will develop cancer; it would be interesting to see whether members of Enterobacteriaceae or Clostridiaceae are more relatively abundant in the gut microbiome of these patients, and whether a rise in these taxa coincides with cancer development. Future studies are warranted to provide a longitudinal view of the role of the microbiome in PHTS.

Sivan *et al*. (2015) showed that *Bifidobacterium* plays a role in antitumor immunity, and identified *B. longum,* an early colonizer of the infant gastrointestinal tract with the ability to break down human milk oligosaccharides, as one of two beneficial species (Schell *et al*. 2002, Underwood *et al*. 2015). Interestingly, we found increased relative abundance of Bifidobacteriaceae, the parent family for genus *Bifidobacterium*, among fecal samples from PHTS patients with no history of cancer. It is tempting to speculate that this family may play a protective role against development of cancer in PHTS patients and perhaps the broader population.

The predicted enrichment of the folate biosynthesis pathway among fecal samples from cancer patients is an interesting finding. Recent studies have linked excessive microbial folate production to a shortened lifespan in *Caenorhabditis elegans,* showing that metformin abrogates this effect by altering microbial folate metabolism (Virk *et al*. 2012, Cabreiro *et al*. 2013). There are many shared pathways in cancer and aging, and anti-folate medications are a mainstay of many cancer chemotherapy regimens. Given our findings, in the context of these associations, it is plausible that microbial folate biosynthesis could be a mediator of cancer risk in individuals with PHTS. Future studies are needed to better elucidate a potential role for microbial folate production in carcinogenesis.

While we found no major shifts in alpha diversity or structural segregation in oral wash samples, we did identify increased relative abundance of *Coriobacteriaceae*, which has been associated with increased non-HDL cholesterol levels in the hamster colon (Martínez *et al*. 2009), among samples from PHTS cancer patients compared to non-cancer patients, and relatively decreased *Gammaproteobacteria*, including genus *Moraxella*, which contains several opportunistic pathogens (Blakeway *et al*. 2017).

The microbiome is a new and rapidly expanding field, with recent advances in DNA sequencing technologies allowing us to move beyond the constraints of culture-dependent techniques to comprehensively examine microbial communities associated with various states of human health and disease. To our knowledge, this is the first study investigating the microbiota of individuals with PHTS or any other inherited cancer syndrome. PHTS patients represent an ideal study population, because, due to their dramatically elevated risks of cancer, we may have a greater chance of detecting a modifiable microbiota contributor that would otherwise be too subtle to detect in the general population.

The major limitations of this pilot study were its small sample size and failure to age- and sex-match cancer and non-cancer patients. A larger, matched study will have greater power to minimize the effect of these confounding factors and detect subtle differences in the microbiota of these groups. Finally, we were limited by the mode of sample collection in this study. Because PHTS is a rare disorder and study patients are spread throughout the world, sample collection kits were shipped to subjects, resulting in variable sample transit time. Importantly, there were no significant differences in antibiotic use over the past 12 months between patients with and without cancer.

While we set out to generate the hypothesis that PHTS-related microbiota could modify cancer status, we did not design our study to analyze microbiome differences between PHTS individuals and controls without *PTEN* mutations. Neither did we design out study to analyze microbiome differences between PHTS with cancer and the general population with sporadic cancer. These hypotheses are worthy and our pilot data should provide a useful platform to inform such future studies. We did intend to compare PHTS with autism spectrum disorder and neurotypical PHTS. However, we were only able to accrue 6 of the latter, all of whom were children, compared to neurotypical PHTS, who were all adults, and we chose to exclude the autism dataset.

From our pilot study, we have generated the hypotheses that there are distinct gut microbial communities in individuals with PHTS with compared to without component cancers, providing initial evidence that differences in gut microbial composition may inform phenotypic outcome in PHTS patients. While these findings are preliminary and require validation in a larger cohort, they suggest that the microbiota may have potential as a diagnostic and/or therapeutic target in the development of cancer in individuals with PHTS, with implications for cancer in the general population. We believe our observations may be sufficient to generate a hypothesis-driven expanded study to reveal differences in microbial composition and diversity not detected by this exploratory study, and to further explore sub-hypotheses suggested by the findings of this pilot study; for example, a potential relationship between the gut microbiota of PHTS patients, non-ovarian estrogen levels and cancer risk or investigate other potential variables affecting microbiota composition, such as seropositivity for common viruses such as HSV, often reactivated in patients with cancer (Djuric *et al*. 2009). In sum, therefore, investigation of the microbiome as a modifier of phenotype in those with heritable cancer syndromes, such as PHTS, is feasible and might show promise from risk assessment to medical management points of view.

## Supplementary Material

Supporting Figure 1

Supporting Figure 2

Supplementary Material 1

Supporting Table 1

## Declaration of interest

The authors declare that there is no conflict of interest that could be perceived as prejudicing the impartiality of the research reported.

## Funding

This work was funded, in part, by the Doris Duke Charitable Foundation Medical Student Clinical Research Mentorship Award (grant #2016080 to V B and C E), P01CA124570 from National Cancer Institute (C E) and U54NS092090 from National Institutes of Health (C E). C E is the Sondra J. and Stephen R. Hardis Endowed Chair of Cancer Genomic Medicine at the Cleveland Clinic and an ACS Clinical Research Professor.

## Author contribution statement

V B and C E contributed to the concept and design of the work, and interpretation of the data. V B, T G, R P, and H A contributed to data acquisition and analysis. The manuscript was drafted by V B and C E. Final figures were created by R P, T G and V B. Figure legends were made by T G and V B. V B, T G, R P, H A and C E critically revised the manuscript and gave final approval.
