# Supplementary figures and images for: The microbiome in PTEN hamartoma tumor syndrome
Source: Endocr Relat Cancer. 2017 Dec 12;25(3):233–43. doi: 10.1530/ERC-17-0442 (PMC5799828; doi:10.1530/ERC-17-0442)

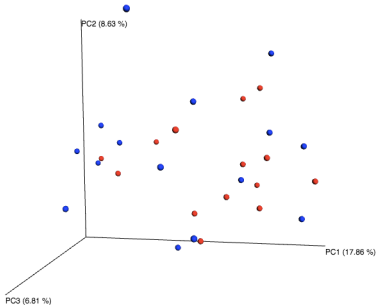

Supplement: Supporting Figure 1 [file erc-25-233-s001.pdf]

A

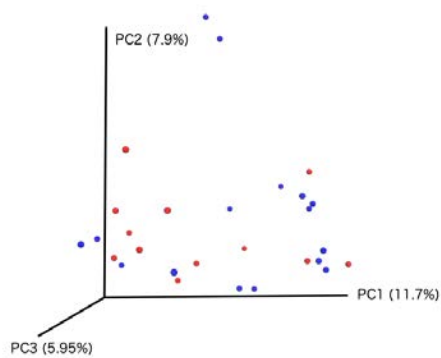

B

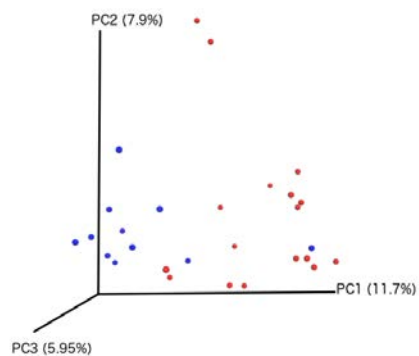

C

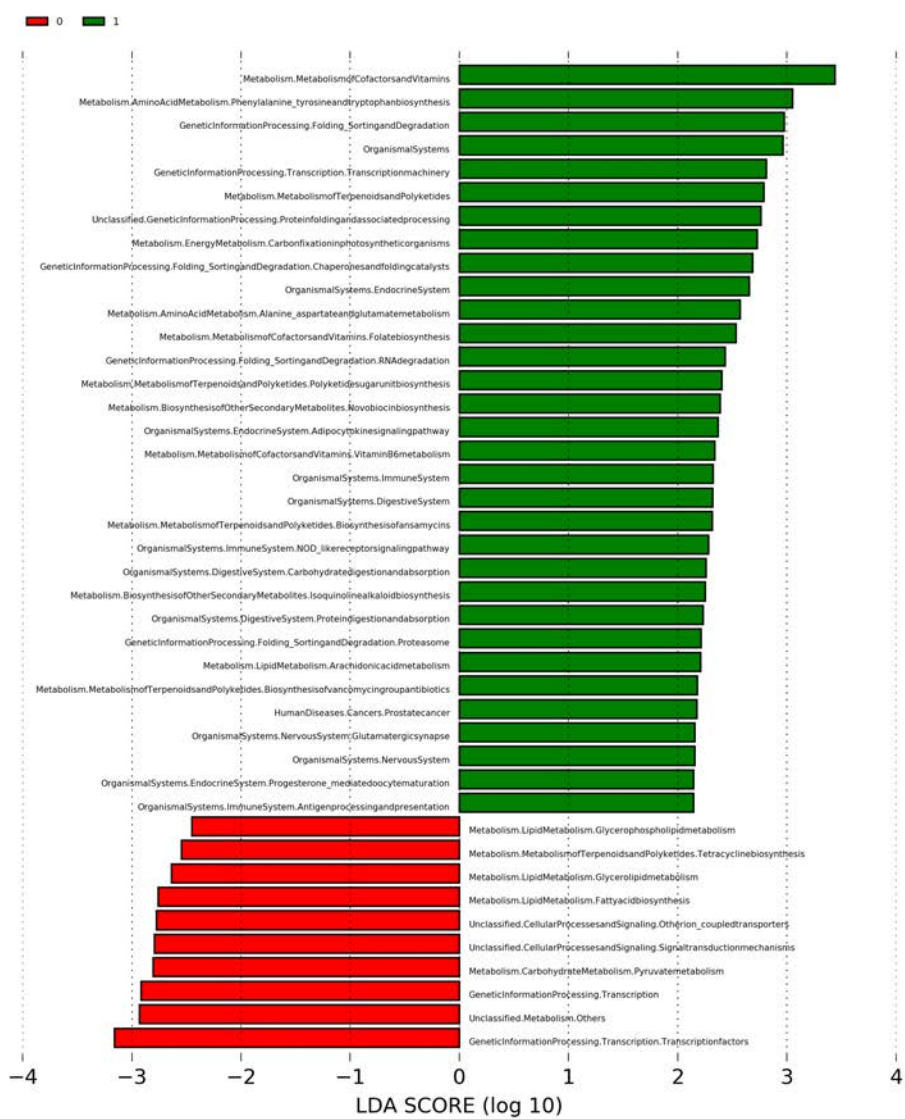

Supplement: Supporting Figure 2 [file erc-25-233-s002.pdf]
