# Supplementary material for: The microbiome in PTEN hamartoma tumor syndrome
Source: Endocr Relat Cancer. 2017 Dec 12;25(3):233–43. doi: 10.1530/ERC-17-0442 (PMC5799828; doi:10.1530/ERC-17-0442)
Supplement: Supplementary Material 1 [file erc-25-233-s003.pdf]

PHTS Microbiome Study Questionnaire

Age (years): _________

Sex (check one):
☐ Male
☐ Female
☐ Other (specify): ________________

BMI
Height (in): ___________
Weight (lbs): ___________

Race/ethnicity (check all that apply):
☐ White non-Hispanic/Latino
☐ White Hispanic/Latino
☐ Black or African-American
☐ Asian or Pacific Islander
☐ Native American or Alaskan Native
☐ Other (specify): __________________________

Smoking status
☐ Never smoker
☐ Former smoker
- o _____ packs/day, _____ years

☐ Current smoker
- o _____ packs/day, _____ years

Do you use probiotics regularly (3 or more times per week)? ☐ Yes ☐ No

Alcohol consumption
☐ Never
☐ Occasional
- o _____ drinks per ☐day ☐week ☐month

☐ Social
- o _____ drinks per ☐day ☐week ☐month

☐ Frequent
- o _____ drinks per ☐day ☐week ☐month

Last oral antibiotic or antifungal use (>1 day):
☐ <1 month ago
☐ 1-6 months ago
☐ 6-12 months ago
☐ >12 months ago

Do you use laxatives regularly (3 or more times per week)? ☐ Yes ☐ No
If Yes, last laxative use:
☐ ≤ 7 days ago
☐ >7 days ago

Diet (check one):
☐ American/Western
☐ Mediterranean
☐ Vegetarian
☐ Vegan
☐ Gluten-free
☐ Low-carb/Atkins
☐ Paleo
☐ Country-specific (Country: _____________)
☐ Other: _____________________

Oral hygiene
How many times per day do you brush your teeth?
☐ Once or less ☐ Twice ☐ 3 or more times
When did you last eat before the oral rinse?
☐ < 1 hour ☐ 1-4 hours ☐ More than 4 hours
When did you brush your teeth before the oral rinse?
☐ < 1 hour ☐ 1-4 hours ☐ More than 4 hours

Past medical history (check all that apply):
☐ Cancer (if yes, please specify): _________________
____
- o Date of diagnosis (year): ___________________

____

☐ Recent hospitalization
- o If yes, how long: _________ ☐days ☐weeks
- o When (month/year): ________________
- o Reason? ______________________

☐ ICU stay
- o If yes, how long: _________ ☐days ☐weeks
- o When (month/year): ________________
- o Reason? _______________________

☐ Recent UTI (if yes, when? ___ ☐weeks ☐mos. ago)
☐ History of kidney stones

☐ Neurological impairment or spinal cord injury with persistent symptoms
Please describe: ________________________
☐ Bladder or GI surgery
Please specify: ________________________
☐ Ulcerative colitis or Crohn's disease
☐ Diverticulitis
☐ C. difficile infection ("C. diff")
Have you had a fecal microbiota transplant?
☐ Yes ☐ No

Birth and developmental history
Were you born via ☐ Vaginal delivery ☐ C-section ☐ Unknown
Were you breastfed as a baby? ☐ Yes ☐ No ☐ Unknown

Have you been given a formal diagnosis of autism or autism spectrum disorder? ☐ Yes ☐ No

Name: ______________________________ CCF#: _____________________________

PHTS Microbiome Study Questionnaire

Please record the following:

- Date __________and time__________ **stool** was collected
  - How long after it was collected was it put in the REFRIGERATOR? ________ ☐ minutes ☐ hours
- Date __________and time__________ **urine** was collected
  - How long after it was collected was it put in the REFRIGERATOR? ________ ☐ minutes ☐ hours
- Date __________and time__________ **oral rinse** was collected
  - How long after it was collected was it put in the REFRIGERATOR? ________ ☐ minutes ☐ hours

** Please DO NOT place the stool, urine, or oral rinse samples in the FREEZER! **

Thank you for your participation in this study!

Name: ______________________________ CCF#: ______________________________
