# Supplementary material for: The microbiome in PTEN hamartoma tumor syndrome
Source: Endocr Relat Cancer. 2017 Dec 12;25(3):233–43. doi: 10.1530/ERC-17-0442 (PMC5799828; doi:10.1530/ERC-17-0442)
Supplement: Supporting Table 1 [file erc-25-233-t001.pdf]

ERC-17-0442R1

The Microbiome in *PTEN* Hamartoma Tumor Syndrome

Supplementary Table 1: Mutations of all subjects

Subject	Mutation
PC1	c.865_871del7 (p.Gly289Lysfs)
PC2	c.164+1G>T
PC3	c.334insG (p.Leu112Argfs*3)
PC4	c.585dupT (p.His196Serfs*6)
PC5	c.697C>T (p.Arg233Ter)
PC9	c.686C>G p.(Ser229*)
PC10	c.1003C>T (p.Arg335Ter)
PC11	c.46T>C (p.Tyr16His)
PC13	c.1061C>A (p.Pro354Gln)
PC14	E2 del (c.80-?_c.164+?del)
PC15	c.1003C>T (p.Arg335Ter)
PC16	c.275A>C (p.Asp92Ala)
PC17	E6 dup(c.493-?_c.634+?dup)
PC19	c.210-1G>A
PC20	c.112C>T (p.Pro38Ser)
PC25	c.195insT (p.Lys66*)
PC30	c.800dupA (p.Lys267fs)
PN1	c.437T>G (p.Leu146Ter)
PN2	c.219_222dupAAGA (p.His75Lysfs*4)
PN3	E2del (c.80-_164+_del_)
PN4	c.955_958delACTT (p.Thr319Ter)
PN5	c.165-2A>G
PN6	c.388C>T (p.Arg130Ter)
PN7	c.97_99delATT (p.Ile33del)
PN8	c.44ins16bp
PN12	c.1-?_1212 +?del
PN18	c.542T>C (p.Leu181Pro)
PN21	c.103A>G (p.Met35Val)
PN22	c.46dupT (p.Tyr16Leufs*28)
PN23	c.892 C>T (p.Gln298Ter)
PN24	c.511C>T (p.Gln171*)
PN29	c.517C>T (p.Arg173Cys)

Mutations are provided in standard mutation nomenclature.

PC: *PTEN* Cancer

PN: *PTEN* Non-cancer
